# An Incidental Finding of Idiopathic Hypertrophic Pachymeningitis: A Case Report

**DOI:** 10.7759/cureus.52087

**Published:** 2024-01-11

**Authors:** Divya Venkat, Keerti Vajrala, Hiral Amin, Robert Jensen

**Affiliations:** 1 Medicine, College of Osteopathic Medicine, Kansas City University of Medicine and Biosciences, Kansas City, USA; 2 Neurology, Menorah Medical Center, Kansas City, USA

**Keywords:** neurologic disorder, dural biopsy, dural thickening, ihp, temporal meningioma, mri brain, inflammation of dura mater, asymptomatic meningitis, idiopathic hypertrophic cranial pachymeningitis, hypertrophic pachymeningitis

## Abstract

In this case report, we discuss and explore the clinical, laboratory, and imaging findings, as well as the treatment options and follow-up measures, in an 83-year-old patient with idiopathic hypertrophic pachymeningitis (IHP), a rare disorder characterized by fibrosing, hypertrophic inflammation that thickens the dura mater. An 83-year-old female with a medical history of hypertension and hyperlipidemia presented with speech arrest and was taken to the emergency department, where she received a stroke code, a CT scan, and an MRI. The MRI results showed a temporal lobe meningioma and a pan-cranial pachymeningitis encasing the entire brain and cerebellum and extending into the upper cervical spine. Multiple unsuccessful attempts at a lumbar puncture were made, so a dural biopsy specimen was obtained, which revealed no malignant process. A cerebral spinal fluid specimen (CSF) from the biopsy showed minimal white blood cells (WBCs) which ruled out infection. Idiopathic hypertrophic pachymeningitis was the given diagnosis based on the apparent MRI findings. The patient was treated in the hospital for four days with IV methylprednisolone and discharged on oral methylprednisolone for four to six weeks.

## Introduction

Idiopathic hypertrophic pachymeningitis (IHP) is a rare disease first described by Jean-Martin Charcot in the 19th century in the context of syphilis and tuberculosis and later classified as a fibrosing, hypertrophic inflammatory process that thickens the dura mater that is either idiopathic or secondary to a wide variety of conditions such as rheumatoid arthritis, syphilis, cancer, tuberculosis, and infections [1]. In several cases, IHP presents clinically with a headache and cranial nerve defects but can also be completely asymptomatic. The exact etiopathogenesis of this condition is still completely unknown. It has often been suspected to be an autoimmune phenomenon. Alternately, it might occur as a direct or indirect consequence of a triggering infection or of an infiltrating neoplasm. A dural biopsy is essential to exclude secondary causes of pachymeningitis [2]. Idiopathic hypertrophic pachymeningitis has been shown to respond to corticosteroids; however, there is a high rate of recurrence when weaned or withdrawn over time [3, 4]. As a distinct disease, IHP is certainly a rare disorder of diverse etiology. Here we present a case of IHP confirmed as a diagnosis through exclusion and extensive MRI scans, treated with corticosteroids.

## Case presentation

The patient, an 83-year-old Caucasian female, presented to the emergency department after an episode of cognitive lapse manifesting as a speech arrest during a conversation with her husband. Her medical history included hypertension and hyperlipidemia. The patient fell mute although she could still understand her husband. She endorsed a slurred speech lasting for 20-30 minutes. Subjectively, she felt that she "just could not get the words out." The patient was brought to the emergency department by ambulance, where this event was hypothesized to represent a transient ischemic attack (TIA). A tele-stroke code was initiated at this time. The National Institute of Health Stroke Scale score in the emergency room was zero. The patient had no apparent neurological deficits. In the course of this video-based stroke evaluation, the patient had CT-based imaging that was largely unremarkable overall. A single-photon emission computerized tomography (SPECT-CT) perfusion revealed no ischemic deficits. Anatomic CT angiography of the brain, neck, and aortic arch revealed no significant stenosis, aneurysm, or vascular anomaly. There was, however, the adventitious finding of a mass at the base of the tongue.

Thrombolytics were not administered, and the patient was admitted for a follow-up MRI scan. A non-contrast MRI scan revealed an ovoid or almond-shaped extra-axial mass along the medial margin of the inferior left temporal lobe without significant mass effect or surrounding vasogenic edema. Subsequent, contrast-enhanced MRI revealed well-marginated, diffusely-enhancing masses extending from that just described adjacent to the mesial temporal lobe to the orbit apex along the sphenoid wing to the clivus and then inferiorly into the left pterygopalatine fossa. There was no apparent parenchymal or bony invasion. This well-marginated, extra-axial, diffusely enhancing tumor was consistent with a meningioma.

**Figure 1:**
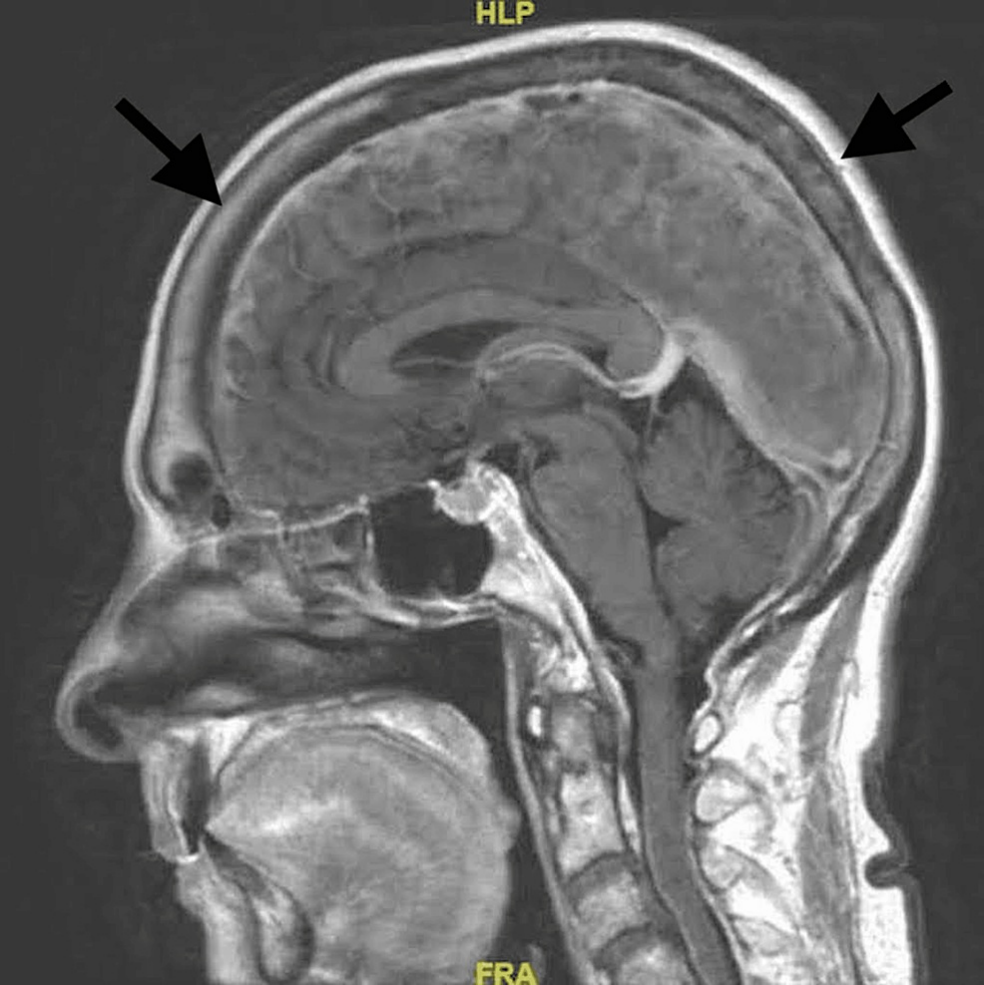
An MRI of the brain (sagittal) T1 FLAIR with contrast Arrows indicate diffuse pachymeningeal thickening that extends into the cervical spine. FLAIR: fluid-attenuated inversion recovery

Even more apparent than the preceding findings, however, was a pan-cranial pachymeningitis completely encasing the entire brain and cerebellum that was very unusual to accompany a typical localized meningioma. The MRI results stated a diffuse pachymeningeal thickening measuring 6 mm on the left and 5 mm along the right cerebral convexity (Figure 1).

**Figure 2:**
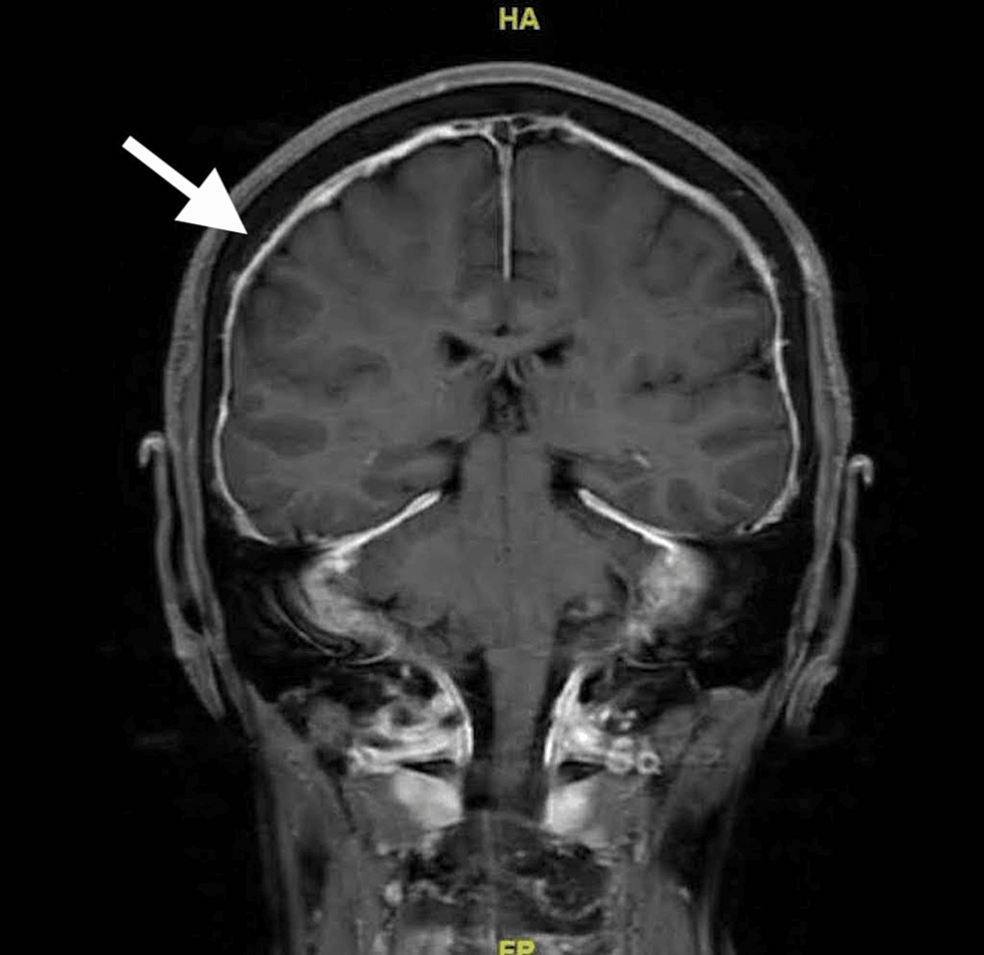
An MRI of the brain (coronal) T1 FLAIR with contrast FLAIR: fluid-attenuated inversion recovery

Pachymeningeal thickening was noted along the prepontine cistern and posterior fossa (Figure 2).

The dural thickening appeared to extend into the upper cervical spine. Consideration was given to lymphoma versus inflammatory disorders such as sarcoidosis, IgG4-related hypertrophic pachymeningitis, Behçet's disease, and dural metastasis. Given its thickness and asymmetric mass-like appearance, intracranial hypotension was unlikely. The patient remained completely asymptomatic at this hospital. She made no complaints, and she had no apparent neurological deficits.

There were multiple attempts to perform a lumbar puncture, all of which failed to produce adequate cerebrospinal fluid (CSF) for analysis. In an attempt to rule out other causes, the patient received a right frontotemporal craniotomy open dural biopsy, which reported dural tissue with minimal acute and chronic inflammation, hemosiderin pigment, calcifications, and no malignant process identified. This was reviewed by two board-certified pathologists. A CSF specimen was also acquired during the biopsy, which showed a WBC count of 10/mm^3, an RBC count of 799/mm^3, a protein count of 108 mg/dL, and a glucose count of 50 mg/dL, as shown in Table 1.

**Table 1:** Dural cerebrospinal fluid findings

Cell Type	Patient Values	Reference Ranges
White Blood Cells (WBC)	10/mm^3	0–5/mm^3
Red Blood Cells (RBC)	799/mm^3	Nil/mm^3
Protein	108 mg/dL	15–40 mg/dL
Glucose	50 mg/dL	50–80 mg/dL

The high RBC count and low WBC count can be explained as a bloody contaminant in the CSF specimen, which is not uncommon for CSF acquired during an open dural biopsy. Based on these findings, which excluded a malignant or infectious cause of pachymeningitis, the diagnosis of IHP was given. 

After reviewing relevant literature, the patient was treated with methylprednisone 1,000 mg IV every 24 hours for four days in the hospital, which she tolerated well. She was then discharged and started on oral methylprednisone for four to six weeks, 1 mg/kg orally every morning. On follow-up, the patient tolerates her treatment plan well and plans to have a repeat MRI and office follow-up in three months.

## Discussion

In this case, we draw attention to an uncommon asymptomatic occurrence of IHP, confirmed with a review of clinical history, physical, imaging, and cerebrospinal specimens. The patient had a concurrent temporal lobe meningioma, which was concluded to be unrelated to the pachymeningitis due to its localization. However, it is fascinating to note that cases of IHP mimicking meningiomas are present in current literature. In these cases, however, the MRI scan focused on the pseudomeningioma showed moderate to severe peri-regional edema surrounding the tumor [5, 6]. This patient’s meningioma showed no mass effect or surrounding edema, leading to the conclusion that the meningioma was an altogether separate entity, unrelated to the IHP findings.

Only about 64 cases of IHP have been reported in the English medical literature from 1860 to 2010, with a current incidence rate of about 0.949 per 100,000 [3]. Early symptoms can be mild and non-specific, which commonly leads to their misdiagnosis. Failure to diagnose IHP and its progression to severe disease can lead to multi-system effects and total neurological involvement. The majority of cases of IHP initially presented with headache, loss of vision, diplopia, papilledema, cranial nerve involvement, ataxia, and seizures [7]. Cranial nerves commonly involved include the optic nerve (II), oculomotor nerve (III), trochlear nerve (IV), abducens nerve (VI), and facial nerve (VII), hence the predominantly visual symptoms are seen in many IHP patients [3]. However, this patient denied any symptoms that could be related to this pachymeningitis other than a sudden episode of speech arrest that could be associated with the underlying meningioma that was found on the MRI.

The etiology of hypertrophic pachymeningitis is complex, and in this particular case, a differential diagnosis of rheumatoid arthritis, tuberculosis, syphilis, IgG4-related diseases, and lymphoma were all considered before classifying this patient with idiopathic hypertrophic pachymeningitis [1,3,7]. The patient in this case did not have a medical history of any underlying autoimmune disease, infection, or systemic diseases, and no such findings were found on a thorough history and laboratory examination.

For the treatment of hypertrophic pachymeningitis, etiologic or symptomatic treatment is typically started first if there is an underlying infection, malignancy, or systemic disease. In IHP, since there is no known cause, corticosteroids are the first choice in treatment [8]. Corticosteroid treatment can help alleviate symptoms and target the inflammation of the dura. Additional immunosuppressive agents can be used if corticosteroid use is ineffective over time [8]. A study done in Japan showed that 87.2% of the 94 patients treated with corticosteroids had a complete resolution of their symptoms. For patients who did not respond to corticosteroids alone, a combination of immunosuppressive agents alleviated symptoms in 92.6% of the patients [3]. Some patients require surgery if all other treatment options fail. The prognosis of IHP varies depending on the speed at which treatment is initiated. It is shown that early treatment with corticosteroids can significantly reduce neurological and ocular symptoms as well as pain in patients who experience pain with this diagnosis [8]. It is important, as per the current literature, to maintain a continuous treatment of corticosteroids to prevent remission or recurrences. About 50% of cases of IHP relapse after treatment, and the duration can last from a week up to multiple years [3].

## Conclusions

This case highlights a patient who presented for speech arrest, possibly due to a meningioma and was diagnosed with asymptomatic IHP. Idiopathic hypertrophic pachymeningitis is a rare disease with a complex symptom profile, ranging from completely asymptomatic to serious and sudden symptoms. Signs to look out for include headaches, vision loss or changes, ataxia, and cranial nerve palsies. However, this disease can be asymptomatic and may be caught incidentally, as in this case. To confirm the diagnosis, an MRI showing dural thickening should be obtained, with the exclusion of other conditions, with a dural biopsy and laboratory testing. Idiopathic hypertrophic pachymeningitis can be managed with oral corticosteroid therapy, immunosuppressive agents, or surgery and should be carefully followed up and continuously treated.
